# Development of a plasmid stabilization system in *Vibrio natriegens* for the high production of 1,3-propanediol and 3-hydroxypropionate

**DOI:** 10.1186/s40643-021-00485-0

**Published:** 2021-12-14

**Authors:** Ye Zhang, Qing Sun, Yu Liu, Xuecong Cen, Dehua Liu, Zhen Chen

**Affiliations:** 1 Key Laboratory of Industrial Biocatalysis (Ministry of Education), Department of Chemical Engineering, Tsinghua University, Beijing, 100084 China; 2 Tsinghua Innovation Center in Dongguan, Dongguan, 523808 China; 3 Center for Synthetic and Systems Biology, Tsinghua University, Beijing, 100084 China

**Keywords:** *Vibrio natriegens*, 1,3-propanediol, 3-hydroxypropionate, Plasmid maintenance, Antibiotic free, Glycerol

## Abstract

*Vibrio natriegens* is a promising industrial chassis with a super-fast growth rate and high substrate uptake rates. *V. natriegens* was previously engineered to produce 1,3-propanediol (1,3-PDO) from glycerol by overexpressing the corresponding genes in a plasmid. However, antibiotic selection pressure for plasmid stability was not satisfactory and plasmid loss resulted in reduced productivity of the bioprocess. In this study, we developed an antibiotic-free plasmid stabilization system for *V. natriegens*. The system was achieved by shifting the *glpD* gene, one of the essential genes for glycerol degradation, from the chromosome to plasmid. With this system, engineered *V. natriegens* can stably maintain a large expression plasmid during the whole fed-batch fermentation and accumulated 69.5 g/L 1,3-PDO in 24 h, which was 23% higher than that based on antibiotic selection system. This system was also applied to engineering *V. natriegens* for the production of 3-hydroxypropionate (3-HP), enabling the engineered strain to accumulate 64.5 g/L 3-HP in 24 h, which was 30% higher than that based on antibiotic system. Overall, the developed strategy could be useful for engineering *V. natriegens* as a platform for the production of value-added chemicals from glycerol.

**Graphic Abstract:**

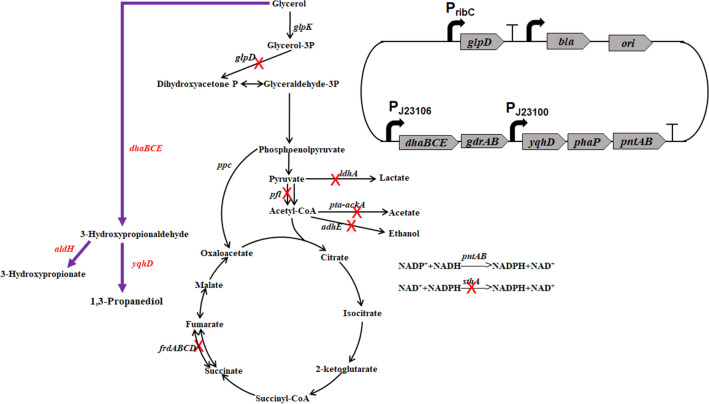

**Supplementary Information:**

The online version contains supplementary material available at 10.1186/s40643-021-00485-0.

## Introduction

The development of economically competitive bioprocesses to produce fuels and chemicals from renewable bioresources is important to achieve the goal of carbon neutrality (Becker and Wittmann 2015; Clomburg et al. 2017). In the past twenty years, metabolic engineering strategies have been widely used to improve the titers and yields of bioproducts, which have significantly increased the economic viability of bioprocesses (Kim et al. 2020; Ko et al. 2020). On the other hand, the productivity of a bioprocess is often limited by the maximum growth rate and/or substrate uptake rate of the employed microorganism (Zhang et al. 2017). Thus, developing fast-growing microorganisms as new industrial chassis could be important for improving the productivity of fermentation processes (Hoffart et al. 2017).

*Vibrio natriegens* is a promising next-generation chassis for industrial biotechnology which has a remarkably short doubling time of less than 10 min in complex media (Weinstock et al. 2016). It can utilize diverse substrates and has an exceptionally high glucose uptake rate under both aerobic and anaerobic conditions (Hoffart et al. 2017), making it an attractive candidate for industrial application. In recent years, different genetic tools and metabolic engineering strategies have been developed to engineer *V. natriegens* to produce value-added chemicals, including alanine, PHB, melanin, 2,3-butanediol, and 1,3-propanediol (Dalia et al. 2017; Hoffart et al. 2017; Lim et al. 2019; Wang et al. 2020; Zhang et al. 2021). In a previous study, we have developed a recombinant *V. natriegens* to efficiently produce 1,3-propanediol (1,3-PDO) from glycerol by introducing and balancing 1,3-PDO synthesis module in a plasmid and systematically optimizing glycerol metabolism and cofactor balance (Zhang et al. 2021). The engineered strain can produce 56.2 g/L 1,3-PDO with a high productivity of 2.36 g/L/h.

Plasmid-based expression systems have been widely used in industrial biotechnology for developing recombinant strains (Kang et al. 2018). Easy manipulation and high-level expression of targeted genes facilitate their wide application. However, the instability of plasmid-based expression systems may hinder their large-scale application in industry. Although antibiotics can be used during fermentation to reduce plasmid loss, the addition of antibiotics to large-scale cultures is expensive and the release of antibiotics to the environment is strongly disfavored (Terrinoni et al. 2018). Moreover, plasmid loss and strain heterogeneity are often observed in large-scale fermentation even with antibiotics addition due to fast cell growth and heterogeneous environments (Rugbjerg and Sommer 2019; Kang et al. 2018). Thus, the development of stable plasmid expression and maintenance systems without using antibiotics is important for industrial application.

Several antibiotic-free plasmid systems have been previously developed to stably maintain plasmids in *Escherichia coli* (Kang et al. 2018; Terrinoni et al. 2018). These systems are normally based on essential gene complementation systems or toxin–antitoxin systems. The implementation of these systems is often very complicated and the application of these systems for the high-level production of chemicals has not been demonstrated. In this study, we discovered that plasmid loss was a key factor affecting the production of 1,3-PDO from glycerol by previously designed *V. natriegens*. A plasmid maintenance system was developed by deleting the *glpD* gene encoding glycerol-3-phosphate dehydrogenase which is essential for glycerol metabolism and by complementing the gene in the expression plasmid. The system was proved to be efficient to increase the plasmid stability and productivity of 1,3-PDO. The system was also successfully applied to develop an efficient *V. natriegens* strain to produce 3-hydroxypropionate (3-HP) from glycerol. To the best of our knowledge, this is the first demonstration of 3-HP production by *V. natriegens*.

## Materials and methods

### Bacterial strains and plasmids

The bacterial strains and plasmids used in this study are listed in Table 1. Plasmids construction was based on the cloning host *E. coli* DH5α. *V. natriegens* VN09 was derived from the wild-type *V. natriegens* ATCC14048 by deleting ten genes related to byproducts formation and metabolic regulation (Δ*adhE*Δ*ldhA*Δ*pfl*Δ*pta-ackA*Δ*arcA*Δ*glpR*Δ*sthA*Δ*frdABCD*Δ*aldAΔaldB*) (Zhang et al. 2021). High-copy plasmid pTrc99a was used for gene overexpression in *V. natriegens*.

**Table 1:** Strains and plasmids used in this study

Strains or plasmids	Description	References
Plasmids		
pTrc99a	High-copy expression plasmid, Amp^R^	Lab collection
pTrc99a-J23106-doy-phaP-pntAB	pTrc99a containing *dhaBCE* and *gdrAB* genes from *K. pneumoniae* under the control of J23106 promoter, an artificial operon containing *yqhD* gene from *E. coli*, *phaP* gene from *Azotobacter sp.*, and *pntAB* genes from *E. coli* under the control of J23100 promoter	Zhang et al. (2021)
pTrc99a-J23106-doy-phaP-pntAB-glpD1	pTrc99a-J23106-doy-phaP-pntAB containing *glpD* gene under the control of *V. natriegens*’ *dnaG* promoter	This study
pTrc99a-J23106-doy-phaP-pntAB-glpD2	pTrc99a-J23106-doy-phaP-pntAB containing *glpD* gene under the control of *V. natriegens*’ *patZ* promoter	This study
pTrc99a-J23106-doy-phaP-pntAB-glpD3	pTrc99a-J23106-doy-phaP-pntAB containing *glpD* gene under the control of *V. natriegens*’ *ribC* promoter	This study
pTrc99a-dhaBCE-aldH	pTrc99a containing *dhaBCE* and *gdrAB* genes from *K. pneumoniae* under the control of J23106 promoter, an artificial operon containing *aldH* gene from *E. coli* and *phaP* gene from *Azotobacter sp.* under the control of J23100 promoter	This study
pTrc99a-dhaBCE-aldH-glpD3	pTrc99a-dhaBCE-aldH containing *glpD* gene under the control of *ribC* promoter	This study
Strains		
* V. natriegens* ATCC14048	Wild-type *V. natriegens*	Lab collection
VN09	*V. natriegens* ATCC14048, Δ*adhE*Δ*ldhA*Δ*pfl*Δ*pta*-a*ckA*Δ*arcA*Δ*glpR*Δ*sthA* Δ*frdABCD*Δ*aldA*Δ*aldB*	Zhang et al. (2021)
VN10	VN9, harboring pTrc99a-J23106-doy-phaP-pntAB	Zhang et al. (2021)
VN10ΔglpD	VN10, Δ*glpD*	This study
VN11	VN09, Δ*glpD*, harboring pTrc99a-J23106-doy-phaP-pntAB-glpD1	This study
VN12	VN09, Δ*glpD*, harboring pTrc99a-J23106-doy-phaP-pntAB-glpD2	This study
VN13	VN09, Δ*glpD*, harboring pTrc99a-J23106-doy-phaP-pntAB-glpD3	This study
VN05	*V. natriegens* ATCC14048, Δ*adhE*Δ*ldhA*Δ*pfl*Δ*pta*-a*ckA*Δ*frdABCD*	This study
VN14	VN05, harboring pTrc99a-dhaBCE-aldH	This study
VN05ΔglpD	VN05, Δ*glpD*,	This study
VN15	VN05ΔglpD, pTrc99a-dhaBCE-aldH-glpD3	This study

### Plasmids construction

To construct plasmid pTrc99a-J23106-doy-phaP-pntAB-glpD1, the native promoter of *dnaG* gene and the *glpD* gene were amplified from *V. natriegens* ATCC14048. The T1 terminator was amplified from plasmid pTrc99a. The three fragments were inserted into the XbaI site of pTrc99a-J23106-doy-phaP-pntAB-glpD1 (Zhang et al. 2021) by the standard protocol of Gibson assembly. The “doy” within the plasmid name indicates the *dhaBCE-gdrAB-yqhD* gene cluster.

Plasmids pTrc99a-J23106-doy-phaP-pntAB-glpD2 and pTrc99a-J23106-doy-phaP-pntAB-glpD3 were constructed by replacing the *dnaG* promoter of plasmid pTrc99a-J23106-doy-phaP-pntAB-glpD1 with the native promoter of *patZ* gene and *ribC* gene of *V. natriegens* ATCC14048 by Gibson assembly.

To construct plasmid pTrc99a-dhaBCE-aldH, the backbone of plasmid pTrc99a-J23106-doy-phaP-pntAB was amplified using PT-F and PT-R primers. The *aldH* gene was amplified from *E. coli* MG1655 using aldH-F and aldH-R primers. The *phaP* gene was amplified from pTrc99a-J23106-doy-phaP-pntAB using phaP-F and phaP-R primers. The three fragments were assembled by using Gibson assembly kits. To obtain plasmid pTrc99a-dhaBCE-aldH-glpD3, a fragment containing *ribC* promoter, *glpD* gene, and T1 promoter was amplified from plasmid pTrc99a-J23106-doy-phaP-pntAB-glpD3 and inserted into the XbaI site of pTrc99a-dhaBCE-aldH. All primers used in this study are listed in Additional file 1: Table S1.

### Strains construction

To delete genes in *V. natriegens* genome*,* the standard protocol combining natural transformation and FLP/FRT recombination system was used as described before (Zhang et al. 2021). A DNA fragment containing the upstream fragment (~ 3000 bp) of the deleted gene, the resistance gene with two FRT loci, and the downstream fragment (~ 3000 bp) of the deleted gene was obtained by overlap extension PCR. The recombinants with selection marker were obtained using natural transformation by following the protocols described by Dalia et al. (2014). To eliminate the selection marker, the selected strain was cultured in LBv2 medium with 1 mM rhamnose at 37 ℃, 200 rpm for 12 h.

### Medium and culture conditions

The LBv2 medium (LB broth supplemented with 200 mM NaCl, 23.14 mM MgCl_2_, and 4.2 mM KCl) is generally used for the culture of *V. natriegens*. Modified VN medium is used for the fermentation of *V. natriegens* to produce 1,3-PDO and 3-HP, containing KH_2_PO_4_ 1.0 g/L, yeast extract 5 g/L, (NH_4_)_2_SO_4_ 5 g/L, NaCl 15 g/L, glycerol 50 g/L, MgSO_4_·7H_2_O 1 g/L, CoCl_2_·6 H_2_O 0.01 g/L, MnCl_2_·4H_2_O 0.01 g/L, FeSO_4_·7H_2_O 0.01 g/L, and vitamin B_12_ 0.005 g/L. The medium was supplemented with 100 μg/mL ampicillin (Amp) to keep the plasmids for the strains without a plasmid stabilization system. For the strains with a plasmid stabilization system, no antibiotics were added.

Fed-batch fermentations were carried out in T&J MiniBox parallel bioreactors with modified VN medium at 37℃. The pH was controlled at 6.5 by automatically feeding 5 M NaOH. The dissolved oxygen was maintained at 10% of air saturation at an aeration rate of 1.0 vvm by adjusting the agitation speed. The feeding solution contains 600 g/L glycerol, 10 g/L yeast extract, 1 g/L MgSO_4_·7H_2_O, 0.01 g/L CoCl_2_·6 H_2_O, 0.01 g/L MnCl_2_·4H_2_O, and 0.01 g/L FeSO_4_·7H_2_O. Another 5 mg/L vitamin B_12_ was added at 12 h.

### Plasmid stability assay

To determine the stability of plasmids, the culture samples were taken at different intervals during the fed-batch fermentation. The samples were diluted with water and plated for single cells on LB plates without antibiotics and incubated overnight at 37 ◦C. Colonies were replica plated onto LB plates containing 100 mg/L ampicillin to check for the presence of the plasmid. The plasmid maintenance ratio was calculated by dividing the colony numbers of LB-ampicillin plates by the colony numbers of LB plates.

### Quantification of biomass and products

Cell concentration was determined by measuring the optical density of 600 nm (OD_600_). High-performance liquid chromatography (HPLC) equipped with an Aminex HPX-87H Column (300 × 7.8 mm) and detection via UV absorption at 210 nm was used to determine the concentration of glycerol, 1,3-PDO, 3-HP, and other organic acids. 5 mM H_2_SO_4_ was used as the mobile phase with a flow rate of 0.8 mL/min (Chen et al. 2014).

### Enzyme assays

The activity of glycerol-3-phosphate dehydrogenase was determined by the method as described by Weiner and Heppel (1972). The activity of alcohol dehydrogenase YqhD toward 3-hydroxypropionaldehyde (3-HPA) reduction was determined as described by Pérez et al. (2008; Sulzenbacher et al. 2004). The activity of aldehyde dehydrogenase AldH toward 3-HPA oxidation was determined as described by Zhao et al. (2019). All of the measures were repeated for three times.

## Results and discussion

### Plasmid stability assay of a recombinant* V. natriegens* during 1,3-PDO production

**Fig. 1:**
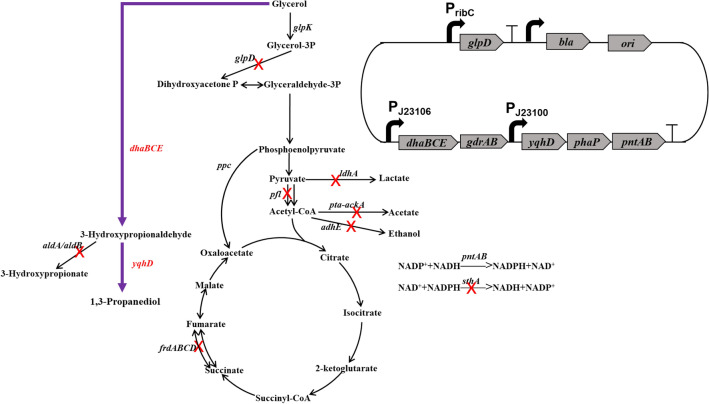
Metabolic engineering strategies for developing *V. natriegens* to produce 1,3-PDO. The deleted genes are shown with cross marks. A heterologous 1,3-PDO synthesis pathway is introduced by overexpressing genes encoding glycerol dehydratase (*dhaBCE*) and its activator (*gdrAB*) and a NADPH-dependent alcohol dehydrogenase (*yqhD*). A plasmid stabilization system is developed by deleting the chromosomal *glpD* gene encoding glycerol-3-phosphate dehydrogenase and expressing the corresponding gene in the expression vector

**Fig. 2:**
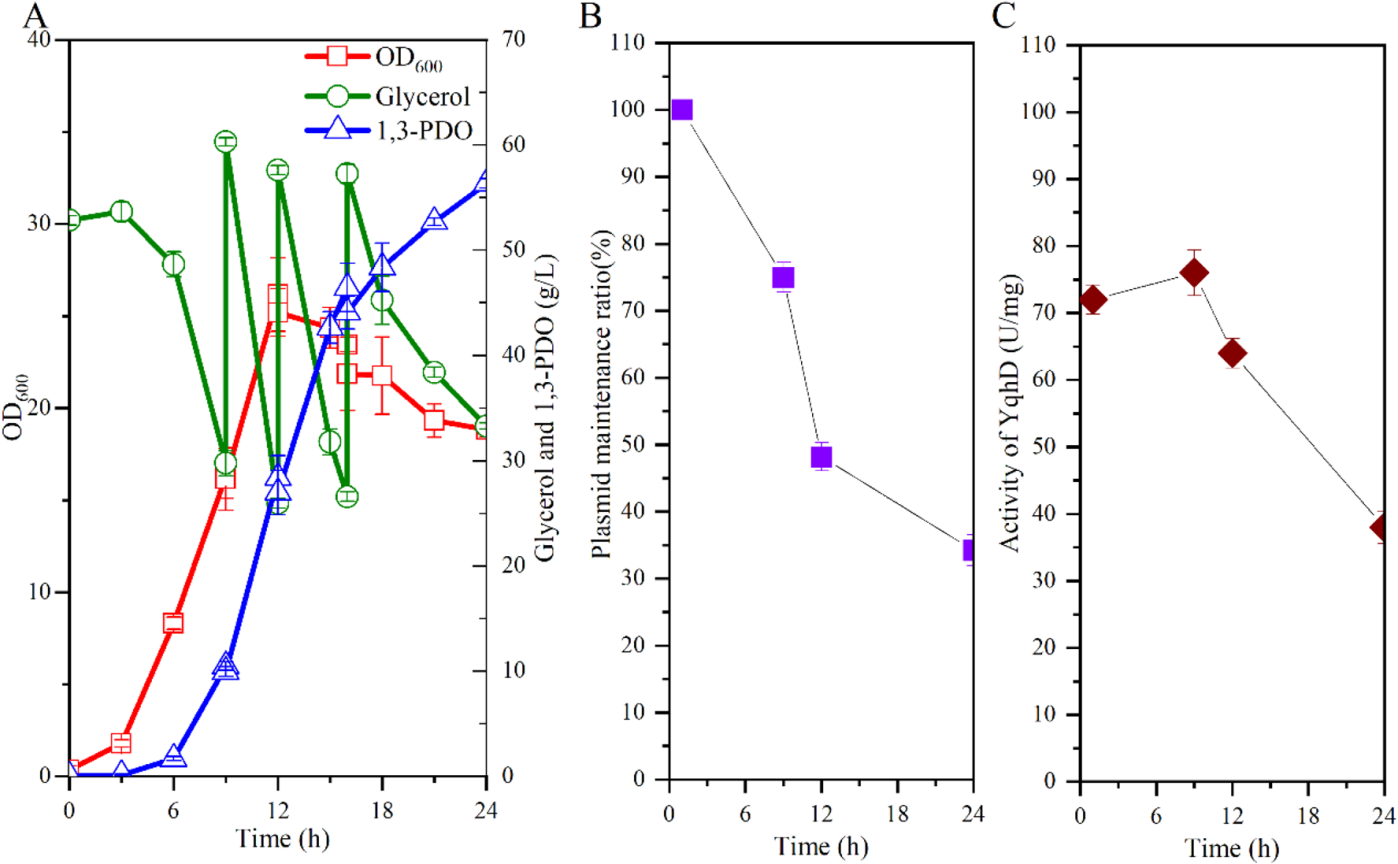
Plasmid stability of *V. natriegens* VN10 during fed-batch fermentation. **A** Fermentation profiles of strain VN10; **B** Plasmid maintenance ratio during fed-batch cultivation; **C** Activity of YqhD during fed-batch cultivation. To maintain the stability of plasmid, 100 mg/L of ampicillin was added in the fermentation medium

In our previous work, a recombinant *V. natriegens* VN10 was constructed to produce 1,3-PDO from glycerol (Zhang et al. 2021). The pathways leading to the formation of main byproducts were all blocked in this strain by deleting *adhE* (ethanol), *ldhA* (lactate), *pfl* (formate), *pta-ackA* (acetate), *frdABCD* (succinate), *aldA, and aldB* genes (3-HP). Moreover, the *arcA* and *glpR* genes encoding two key transcriptional regulators (Nizam et al. 2009) and the *sthA* gene encoding a soluble pyridine nucleotide transhydrogenase were also deleted to increase NADPH availability (Fig. 1). An optimized 1,3-PDO synthesis module was overexpressed in plasmid pTrc99a by placing *dhaBCE* and *gdrAB* genes encoding glycerol dehydratase and its activator under promoter J23106 and *yqhD* gene encoding NADPH-dependent alcohol dehydrogenase under promoter J23100 (Fig. 1). Moreover, the *phaP* gene from *Azotobacter sp.* encoding phasin PhaP and the transhydrogenase genes *pntAB* were also overexpressed in the plasmid to increase the robustness of cell and the intracellular concentration of NADPH, respectively (Fig. 1). PhaP is a polyhydroxyalkanoate granule-associated protein that has been used to protect cells against several kinds of stress, including toxic metabolites, such as ethanol, butanol, and 3-hydroxybutyrate (de Almeida et al. 2007; Mezzina et al. 2017; Zhang et al. 2021). It has also been successfully used to increase the production of 1,3-PDO (Mezzina et al. 2017; Zhang et al. 2021). Strain VN10 can produce 56.4 g/L 1,3-PDO in 24 h with a yield of 0.61 mol/mol glycerol during fed-batch fermentation without the accumulation of other byproducts (Fig. 2A). However, a sharp reduction of the productivity of 1,3-PDO was observed after 15 h (Fig. 2A). Since 1,3-PDO synthesis module was overexpressed in the plasmid and the plasmid was very large (~ 13.7 kb), we supposed that instability of the plasmid may be one important reason related to the reduced productivity. To test this hypothesis, we took samples at different time intervals during fed-batch fermentation and measured the plasmid maintenance ratios. As shown in Fig. 2B, a high ratio of plasmid loss was observed during fed-batch fermentation. Although antibiotic (100 mg/L ampicillin) was added in the medium, 51.8% and 65.8% of cells lost plasmid at 12 h and 24 h. We also measured the activity of NADPH-dependent alcohol dehydrogenase (YqhD) during fed-batch fermentation. As shown in Fig. 2C, the activity of YqhD was also significantly reduced after 12 h, suggesting that the reduced productivity of 1,3-PDO may be related to plasmid loss. To reduce plasmid loss, we tried to add ampicillin at different time intervals during fed-batch fermentation, which, however, did not improve the plasmid maintenance ratios or the productivity of 1,3-PDO (data not shown).

### Designing a plasmid maintenance system to improve 1,3-PDO production

**Fig. 3:**
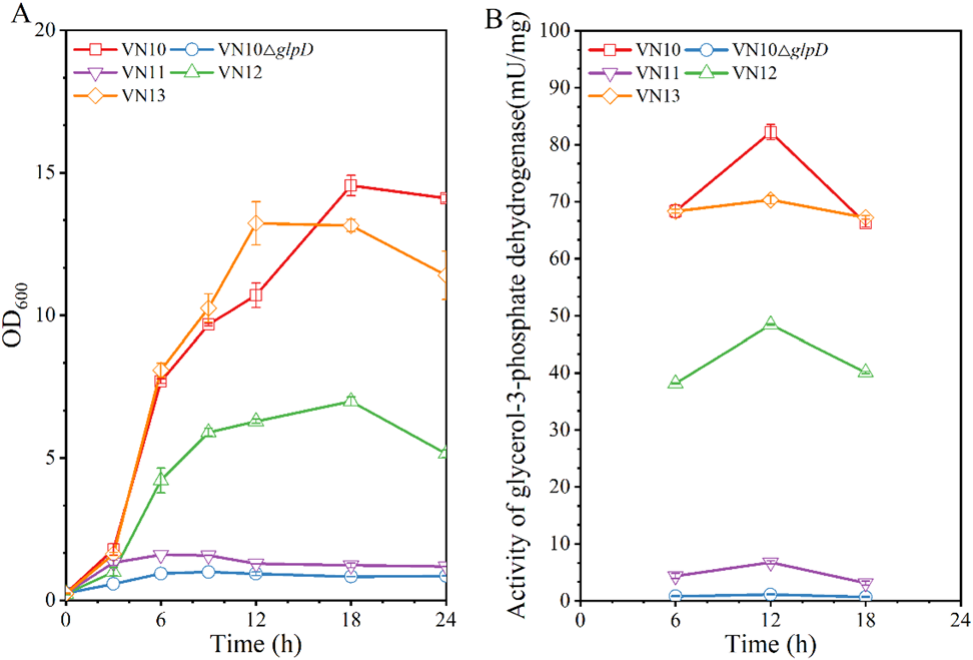
Development of a plasmid maintenance system by adjusting the promoter of *glpD* gene in the expression vector. **A** Cell growth; **B** Activity of glycerol-3-phosphate dehydrogenase during the shake flask cultivation

To increase plasmid stability during fed-batch fermentation of *V. natriegens*, we attempted to design a plasmid maintenance system by coupling substrate consumption, cell viability, and product formation in the expression plasmid. *V. natriegens* has only one glycerol consumption pathway which is composed of glycerol kinase (*glpK*) and glycerol-3-phosphate dehydrogenase (*glpD*) (Zhang et al. 2021). Our previous study showed that the expression of *glpK* gene was subjected to complex genetic regulation (Zhang et al. 2021). To avoid significant perturbation of cellular metabolism, we select the *glpD* gene as a target for designing a gene complementation system. The chromosomal *glpD* gene was deleted and expressed in the expression plasmid pTrc99a-J23106-doy-phaP-pntAB. Knockout of *glpD* gene abolishes the growth of *V. natriegens* VN10 in M9 minimal medium with glycerol as the sole carbon source. *V. natriegens* VN10△*glpD* showed marginal growth in fermentation medium due to the presence of yeast extract but did not consume glycerol (Fig. 3A). To recover the activity of glycerol-3-phosphate dehydrogenase, the *glpD* gene was inserted into plasmid pTrc99a-J23106-doy-phaP-pntAB with different promoters. With the consideration of plasmid number of pTrc99a, three constitutive promoters from *V. natriegens* with varied strength (1% ~ 10% of the expression level of chromosomal *glpD* gene) were selected, including the native promoters of *dnaG* gene, *patZ* gene, and *ribC* gene (Lee et al. 2019; Zhang et al. 2021). As shown in Fig. 3A and B, when the promoter of *ribC* gene was used to express plasmid-bearing *glpD* gene, strain VN13 recovered normal cell growth and showed similar activity of glycerol-3-phosphate dehydrogenase compared to strain VN10.

**Fig. 4:**
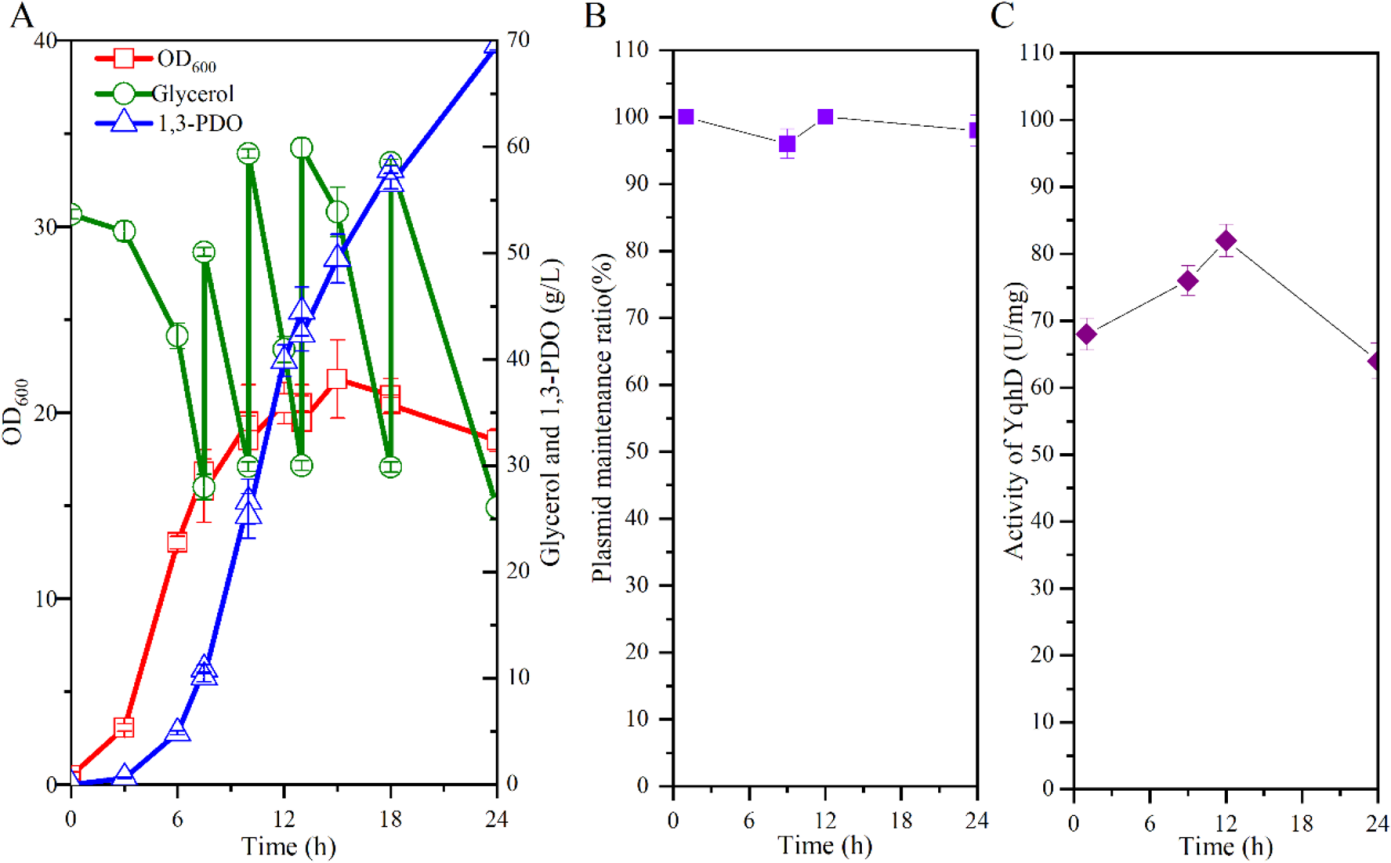
Increasing the production of 1,3-PDO by strain VN13 with a plasmid stabilization system in fed-batch fermentation. **A** Fermentation profiles of strain VN13; **B** Plasmid maintenance ratio during fed-batch cultivation; **C** Activity of YqhD during fed-batch cultivation. No antibiotics were added in the fermentation medium

The performance of strain VN13 was tested in fed-batch fermentation without adding antibiotics. As shown in Fig. 4A, strain VN13 produced 69.5 g/L 1,3-PDO in 24 h, with a productivity of 2.90 g/L/h and a yield of 0.62 mol/mol. The productivity of 1,3-PDO was 23% higher than that obtained by strain VN10 based on the antibiotic selection system. The expression plasmid in strain VN13 was almost completely maintained during the whole fed-batch fermentation (Fig. 4B), indicating that the designed system was efficient to couple cell viability with plasmid maintenance. The activity of YqhD was also retained at a high level during the whole fed-batch fermentation (Fig. 4C), suggesting that increased plasmid stability is helpful to enhance pathway efficiency and 1,3-PDO production. The yield and productivity of 1,3-PDO by strain VN13 are higher than most natural 1,3-PDO producers (Lee et al. 2015, 2018; Sun et al. 2018;). Thus, the developed plasmid maintenance system was shown to be effective and robust for increasing 1,3-PDO production under antibiotic-free cultivation, which is strongly important for large-scale fermentation in industry. Compared to strain VN10, the yield of 1,3-PDO by strain VN13 was not significantly increased probably due to that the flux distribution to glycerol oxidative and reductive pathways was partially altered due to the expression of *glpD* gene in the expression plasmid. It is possible to increase the yield of 1,3-PDO by further optimizing the promoter strength of *glpD* gene in the plasmid. The final titer of 1,3-PDO could also be increased by extending the fermentation time. However, the productivity of 1,3-PDO was significantly reduced after 24 h due to severe cell death (data not shown) although the cells still had a high plasmid maintenance ratio (> 95%). The reasons for the severe cell death are unknown and should be elucidated in the future.

### Application of the plasmid maintenance system for developing a 3-HP hyperproducer

**Fig. 5:**
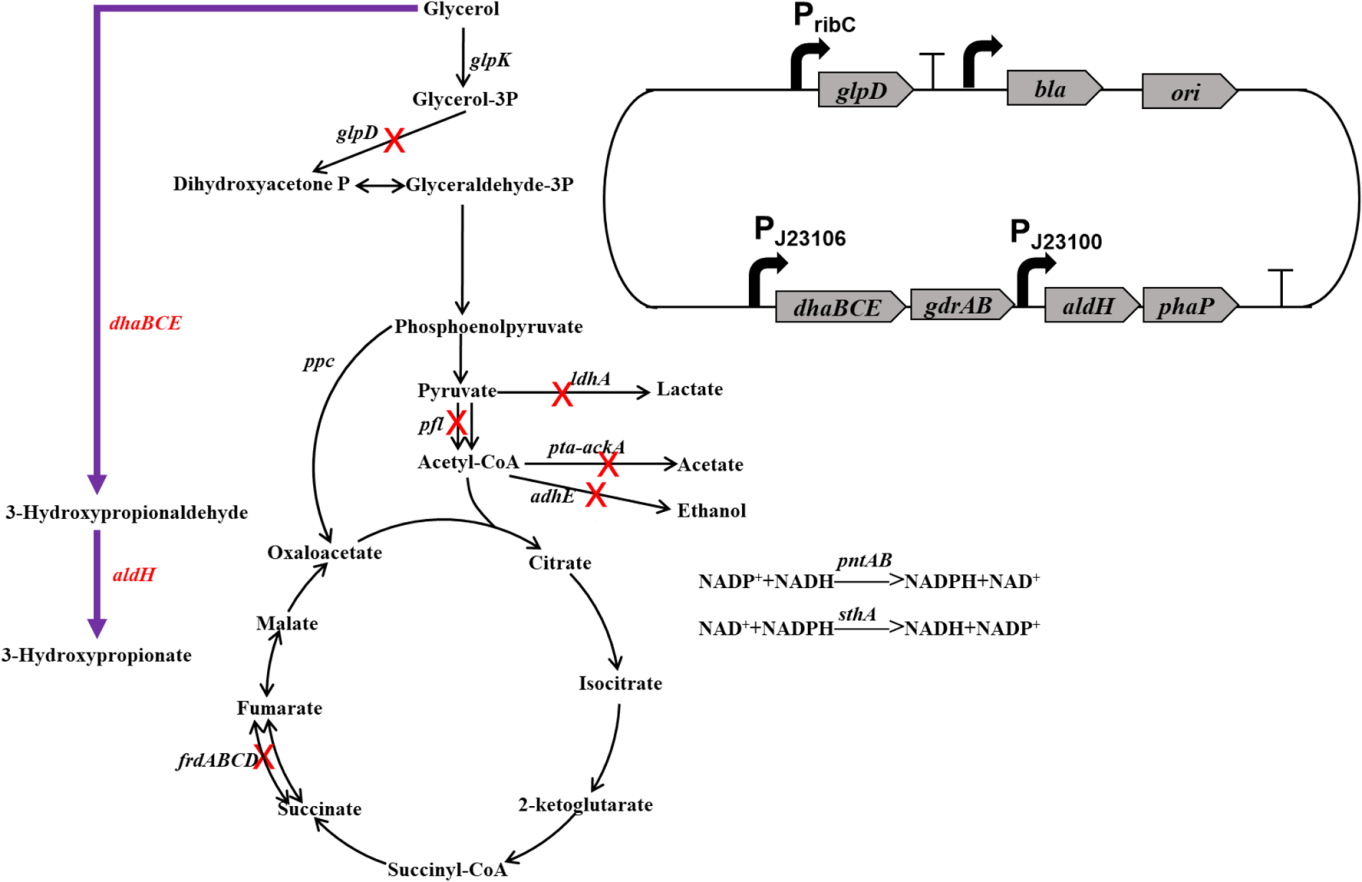
Application of the plasmid maintenance system for developing *V. natriegens* to produce 3-HP. The deleted genes are shown with cross marks. A heterologous 3-HP synthesis pathway is introduced by overexpressing genes encoding glycerol dehydratase (*dhaBCE*) and its activator (*gdrAB*) and a NAD-dependent aldehyde dehydrogenase (*aldH*). A plasmid stabilization system is developed by deleting the chromosomal *glpD* gene encoding glycerol-3-phosphate dehydrogenase and expressing the corresponding gene in the expression vector

**Fig. 6:**
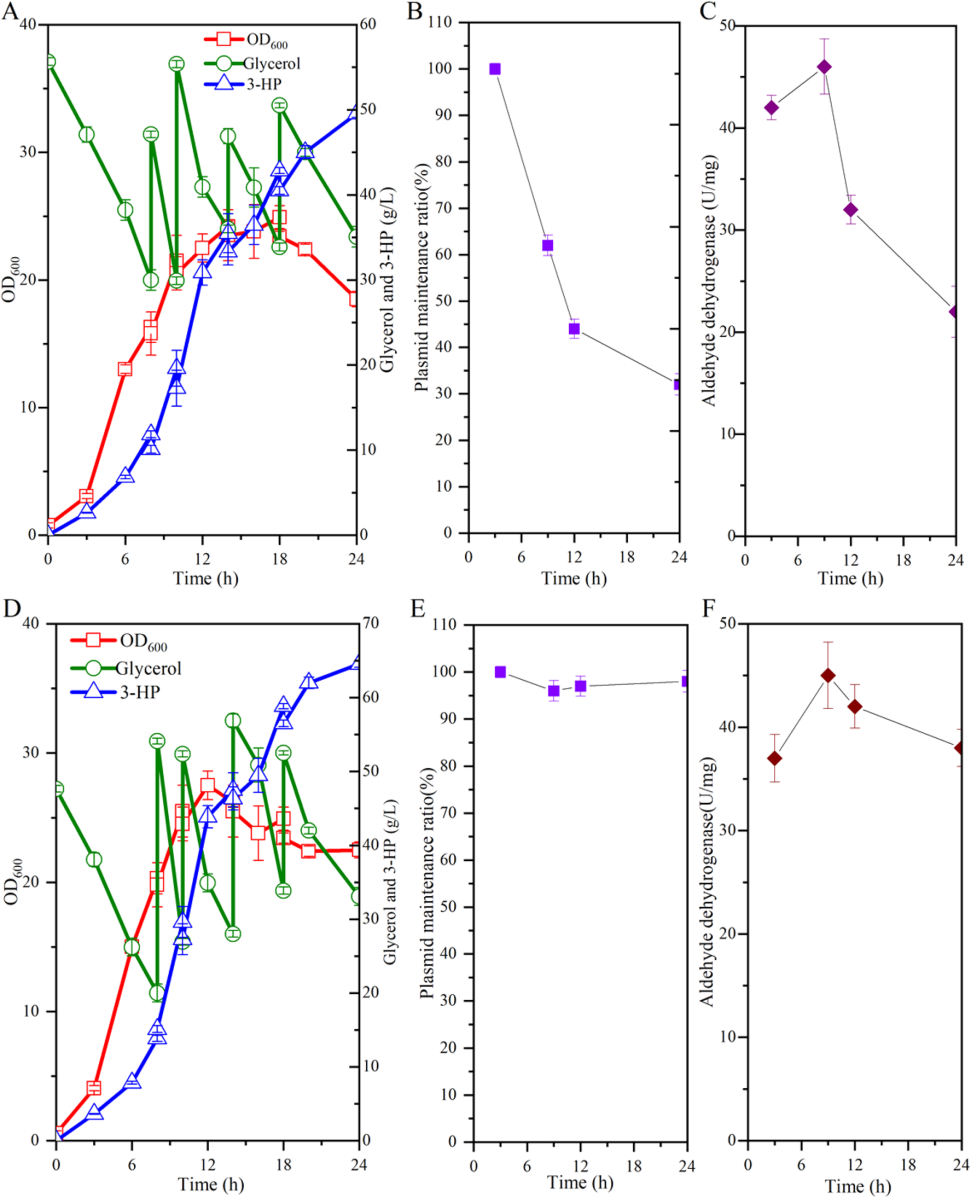
Production of 3-HP by strain VN14 and strain VN15 (with a plasmid maintenance system) during fed-batch fermentation. **A** Fermentation profiles of strain VN14; **B** Plasmid maintenance ratio by strain VN14; **C** Activity of aldehyde dehydrogenase of strain VN14; **D** Fermentation profiles of strain VN15; **E** Plasmid maintenance ratio by strain VN15; **F** Activity of aldehyde dehydrogenase of strain VN15. 100 mg/L of ampicillin was added during the fermentation of strain VN14, while no antibiotics were added during the fermentation of strain VN15

To test the general applicability of the designed plasmid maintenance system, we attempted to implement the system for 3-HP production. 3-HP is an important platform chemical that can be used as a precursor for the synthesis of various chemicals, such as acrylic acid, acrylonitrile, and propiolactone (Chen et al. 2017; Dishisha et al. 2015; Kim et al. 2020). Different microorganisms have been engineered to produce 3-HP, including *E. coli*, *Klebsiella pneumoniae*, and *Corynebacterium glutamicum* (Chen et al. 2017; Kim et al. 2020; Zhao et al. 2019). However, the productivities of 3-HP by all reported microorganisms are lower than 2.5 g/L/h, which can be a limiting factor for its industrial application. Considering its fast growth and high substrate consumption rate, *V. natriegens* could be a potential chassis for 3-HP production. Since the conversion of glycerol to 3-HP is an oxidative process which generates NADH, we constructed a new *V. natriegens* starting strain VN05 by deleting the *adhE*, *ldhA*, *pfl*, *pta-ackA*, and *frdABCD* genes to prevent the accumulate of other byproducts (Fig. 5). The 3-HP synthesis module was firstly established by constructing plasmid pTrc99a-dhaBCE-aldH which overexpressed *dhaBCE* and *gdrAB* genes under promoter J23106 and *aldH* gene encoding an efficient aldehyde dehydrogenase from *E. coli* under promoter J23100 (Jo et al. 2008). The phasin PhaP gene *phaP* was also introduced to increase the strain’s tolerance to 3-HP (de Almeida et al. 2007; Mezzina et al. 2017). By introducing plasmid pTrc99a-dhaBCE-aldH into *V. natriegens* VN05, the generated strain VN14 was able to accumulate 49.5 g/L 3-HP by fed-batch fermentation with the addition of ampicillin, with a productivity of 2.06 g/L/h and a yield of 0.68 mol/mol (Fig. 6A). No other byproducts were detected during the fermentation. However, severe loss of plasmid was also observed during the fed-batch fermentation (Fig. 6B). At 24 h, the plasmid maintenance ratio was only 32% and the activity of aldehyde dehydrogenase was also sharply reduced after 9 h (Fig. 6B and C). Thus, the plasmid maintenance system was also introduced by deleting the chromosomal *glpD* gene and expressing it in plasmid pTrc99a-dhaBCE-aldH-glpD3 under the control of *ribC* promoter. The resulting strain VN15 produced 64.5 g/L 3-HP during the fed-batch fermentation without adding antibiotics, with a productivity of 2.69 g/L/h and a yield of 0.67 mol/mol (Fig. 6D). The expression plasmid was almost completely maintained and the activity of aldehyde dehydrogenase was retained at a high level during the fermentation (Fig. 6E and F). Thus, the developed plasmid maintenance system was also effective for increasing 3-HP production under antibiotic-free cultivation. To the best of our knowledge, the obtained productivity of 3-HP was the highest value reported to date.

## Conclusion

In this study, a plasmid maintenance system was developed to increase the stability of large plasmids in *V. natriegens*. By deleting the chromosomal *glpD* gene and complementing it in an expression vector, the system was shown to be effective to prevent plasmid loss since the *glpD* gene is essential for cell survival and glycerol consumption in the cultivation using glycerol as the sole carbon source. The system was successfully implemented to engineer *V. natriegens* to produce 1,3-PDO and 3-HP from glycerol, enabling the resulting strains to produce 69.5 g/L 1,3-PDO and 64.5 g/L 3-HP in 24 h. The obtained productivities were significantly higher than those by antibiotic selection cultivations. The developed antibiotic-free system provided an important tool for engineering *V. natriegens* as an industrial chassis for the production of chemicals on a large scale. The system can also be modified to produce chemicals from other carbon sources by selecting alternative essential genes.

### Supplementary Information

**Additional file 1: Table S1.** Primers used in this study.

## Data Availability

Not applicable.

## Abbreviations

1,3-PDO: 1,3-Propanediol
3-HP: 3-Hydroxypropionate
OD_600_: Optical density at wavelength 600 nm
HPLC: High-performance liquid chromatography
