# Supplementary material for: Development of a plasmid stabilization system in Vibrio natriegens for the high production of 1,3-propanediol and 3-hydroxypropionate
Source: Bioresour Bioprocess. 2021 Dec 14;8(1):125. doi: 10.1186/s40643-021-00485-0 (PMC10992974; doi:10.1186/s40643-021-00485-0)
Supplement: Supplementary file 1 — Additional file 1: Table S1. Primers used in this study. [file 40643_2021_485_MOESM1_ESM.docx]

**Table S1. Primers used in this study**

| **Primer name** | **Sequence** **(5′– 3′)** |
| --- | --- |
| **pTrc99a-J23106-doy-phaP-pntAB-glpD1 construction** | |
| dnaG-F | atcctgacggatggccttttgtgctgcaaacaaattttgtacagacaataat |
| dnaG-R | gcatttttttgtatactcataaaacctaacaaaaaagaaaaatcatgattggtg |
| glpD-F | tttcttttttgttaggttttatgagtatacaaaaaaatgcttccacaactg |
| glpD-R | gaaaatcttctctcatccgccaaaacagccattagcccacttgtgagaggttcac |
| T1-F | gtgaacctctcacaagtgggctaatggctgttttggcggatgagagaa |
| T1-R | tatttagaaaaataaacaaaaagagtttgtagaaacgcaaaaag |
| **pTrc99a-J23106-doy-phaP-pntAB-glpD2 construction** | |
| backbone-F | gttaaaggcaaggaatctgcatgagtatacaaaaaaatgcttccacaactgac |
| backbone-R | ctattatgcggtggtggctcaaaaggccatccgtcaggat |
| patZ-F | atcctgacggatggccttttgagccaccaccgcataatagaaga |
| patZ-R | gcatttttttgtatactcatgcagattccttgcctttaacacga |
| **pTrc99a-J23106-doy-phaP-pntAB-glpD3 construction** | |
| backbone-F | tgaaagcgcaaattcgtctgaaaaggccatccgtcaggatg |
| backbone-R | cagataagtaggtataaacaatgagtatacaaaaaaatgcttccacaactgac |
| Fragment-F | atcctgacggatggccttttcagacgaatttgcgctttcagttg |
| Fragment-R | gcatttttttgtatactcattgtttatacctacttatctgatctctactaaacaacgc |
| **pTrc99a-dhaBCE-aldH construction** | |
| PT-F | ggtatatctccttgctagcactgtaccta |
| PT-R | ggtaaaaaaaccgcgtaatgcaggcatgcacaaataaaacgaaaggctcagtcgaaag |
| aldH-F | taggtacagtgctagcaaggagatataccatgaattttcatcatctggct |
| aldH-R | tcaggcctccaggcttatcca |
| phaP-F | tggataagcctggaggcctgaaaggagatataccatggcgttt |
| phaP-R | ttacgcggtttttttaccag |
| **pTrc99a-dhaBCE-aldH-glpD3 construction** | |
| orf-F | atcctgacggatggccttttcagacgaatttgcgctttcagttg |
| orf-R | acaaaaagagtttgtagaaacgcaaaaaggccatccgtcaggatgg |
|  |  |
